# Spectroscopic Study of the Common Features of Melanins Synthesized from a Diverse Range of Precursors

**DOI:** 10.3390/molecules31203687

**Published:** 2026-10-15

**Authors:** Koen Vercruysse

**Affiliations:** Chemistry Department, Tennessee State University, Nashville, TN 37209, USA; kvercruysse@tnstate.edu

**Keywords:** melanin, absorbance spectroscopy, FT-IR spectroscopy, catechols, serotonin, colloid

## Abstract

Melanins were synthesized using a variety of precursors, some of which are precursors of naturally occurring eumelanin or allomelanin. Purified materials were evaluated using absorbance or FT-IR spectroscopy and compared for their common physical and chemical properties. Absorbance spectra were modeled using an exponential and a novel reciprocal power equation to objectively quantify the appearance of the melanin. The application of the reciprocal power equation was demonstrated to provide an estimate of the band gap width of the melanin material. Both the absorbance and FT-IR spectra of the various melanins showed qualitative similarities despite the chemical diversity of the precursors involved. FT-IR spectroscopy was employed to evaluate the presence of carboxylic acid or carboxylate functional groups and provide evidence of possible oxidative cleavage of aromatic rings during the synthesis of melanin. Through precipitation experiments using Ca^2+^ the physical (in)stability of melanin dispersions was evaluated and their colloidal properties highlighted.

## 1. Introduction

**Figure 1:**
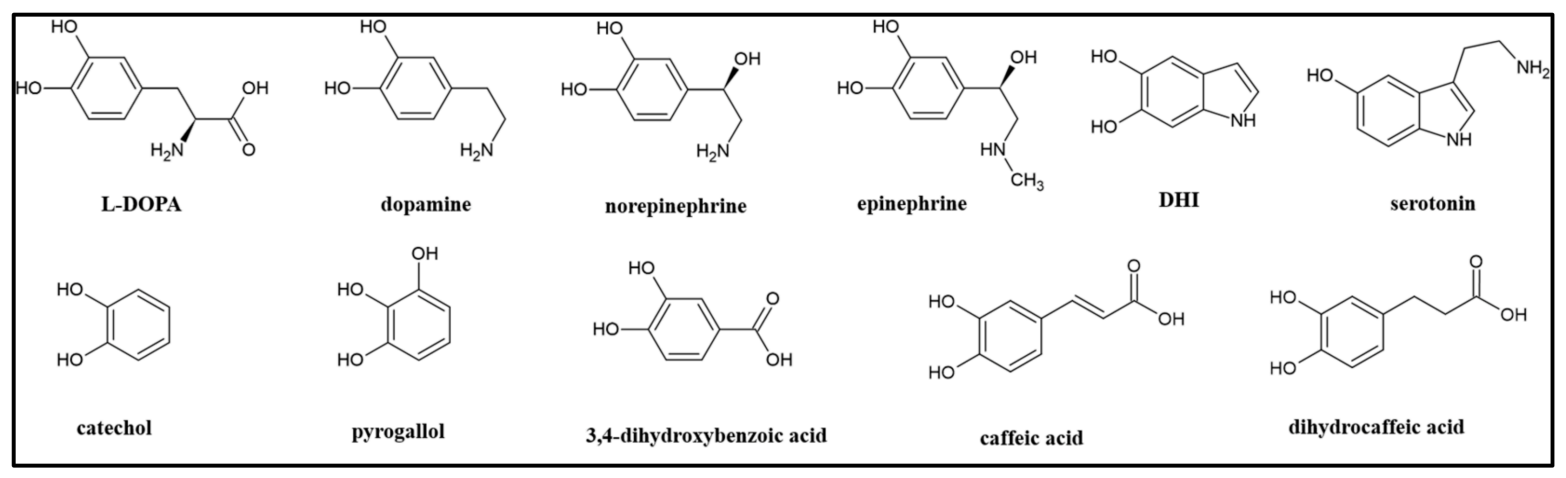
Chemical structures of the precursors used in the synthesis of MNs discussed in this report.

Melanin (MN) is a term used to describe a diverse group of biomolecules present in many groups of living organisms and has been the subject of various reviews. Despite decades of research, MN is still incompletely defined in its chemical structure and review reports on MN offer diverse descriptions of what constitutes MN [1,2,3,4,5,6,7]. In human and other animal species’ physiology two distinct classes of MN are responsible for the coloration of skin and hair: eumelanin (EuMN) and pheomelanin (PhMN) [8,9]. EuMN is typically described as a brown-to-black-colored material built from L-DOPA as the precursor. PhMN is commonly described as a yellow-to-red pigment formed by a combination of L-DOPA and the amino acid cysteine. This distinction between EuMN and PhMN may only be applicable for in vivo biosynthesis. In vitro, the addition of L-cysteine to the air-mediated oxidation of MN precursors in an alkaline environment leads to darker, visually more EuMN-like materials [10]. On the other hand, yellow-to-red materials can be generated in vitro if other precursors, e.g., epinephrine, are used, or may be hiding behind the dark-colored, EuMN material [10,11]. Dopamine and norepinephrine, with or without cysteine, have been described as the precursors of neuromelanin, a MN found in the brains of humans and animals [12]. Plant, fungal and some bacterial MNs are built from nitrogen-free precursors, e.g., catechol, caffeic acid, homogentisic acid, or dihydroxynaphthalene [2,3,13,14]. The experiments described in this report involve MNs synthesized from a wide variety of catecholic (ortho biphenolic) precursors. An exception to this is the use of serotonin in select experiments. Serotonin-related experiments are included in this report for (a) their unique capacity to spontaneously form structural colors, (b) the physiological importance of serotonin, (c) the similarities between the chemical structure of serotonin and the chemical structure of 5,6-dihydroxyindole (DHI), an intermediate in the synthesis of melanin [15], and (d) the precedent set in other reports [16,17]. A striking feature of MNs is their capacity to absorb light in a monotonic, exponentially declining fashion through the ultraviolet (UV), visible (vis) and near infrared (NIR) regions of the electromagnetic spectrum. This phenomenon creates their appearance as dark-colored, EuMN, or light-colored, PhMN, and anything in between [10]. This monotonic absorbance spectrum has been explained in terms of an overlay of the absorbance spectra of individual chemical species present in the material; the so-called chemical diversity model [18,19,20]. An alternative model invokes a combination of geometric order and disorder within MN-aggregated structures, leading to delocalized excitonic effects amongst the oligomeric units making up the aggregates [21]. In other reports it has been argued that the monotonic absorbance spectra observed in MNs are due to charge transfer phenomena [22,23]. An excellent review and discussion on the interaction of MNs with electromagnetic radiation is given by Xie et al. [6]. Despite the fact that most review papers will highlight MN’s insolubility in water or organic solvents, MNs are often described as “soluble” in alkaline, e.g., KOH or NaOH, environments [3,24]. The synthesis process used in our experiments involved the use of Na_2_CO_3_ as the base agent to initiate the reaction and the MNs generated almost always presented themselves as seemingly dissolved in the reaction medium. It has been argued that the MNs thus generated are not dissolved but are dispersions of colloidal, hybrid materials [11,25]. MNs have been described as colloidal particles before and many applications are explored based on these colloidal properties [3,6,14,26,27,28,29,30,31,32,33,34]. In this context it is worth noting that, despite the uncertainties regarding the chemical structure(s) constituting MNs, there seems to be a consensus that MNs are supramolecular structures generated through a multi-step process, proceeding from oligomers to aggregation into proto-particles, and the final, so-called, type A and type B particles [35,36]. The approach in our studies is to use a broad variety of precursors to synthesize MNs and compare their spectroscopic or other properties. Figure 1 shows the chemical structures used as precursors for the synthesis of the MNs discussed in this report.

L-DOPA, dopamine or norepinephrine are considered precursors for EuMN-like materials, while nitrogen-free compounds like catechol or caffeic acid are considered precursors for allomelanin-like materials [1,2]. For reasons outlined above, as cysteine was not added to any of the reactions, no PhMN-like materials were attempted to be synthesized.

MNs were generated through air-mediated oxidation in the presence of Na_2_CO_3_ and were evaluated for their capacity to absorb light from the UV to the NIR region of the electromagnetic spectrum, and their FT-IR spectroscopic properties. In addition, using FT-IR spectroscopy, select materials were compared before and after acidification of the material. The use of different precursors to generate MNs allowed us to explore the spectroscopic features that MNs have in common. This report highlights three important features: (1) the use of two different mathematical equations and their associated parameters to objectively evaluate, quantitatively and qualitatively, the absorbance and appearance of any MN material, (2) the use of FT-IR spectroscopic analyses to evaluate the common features amongst all MNs, and the presence of carboxylic acid or carboxylate functional groups, and (3) the interaction between MNs and Ca^2+^ to evaluate the colloidal properties of the materials.

## 2. Results

### 2.1. Visual Observations on Stagnant Reactions with Serotonin and Others

Reactions involving serotonin were unique amongst all precursors studied thus far. MN formation never yielded a physically stable dispersion but always resulted in extensive precipitations and coating of reaction containers. When kept under stagnant conditions a film formed at the top of the reaction mixture that had a black appearance when viewed perpendicularly. When viewed from a different angle, iridescent color was observed. The color of the film appeared to change as the reaction proceeded. These observations are illustrated in Supplementary Figure S1a–d. After 7 days the mixture was stirred, and significant amounts of precipitation were observed. The mixture was centrifuged, and the colorless supernatant was kept at room temperature for an additional 7 days. Color and film formation appeared anew as shown in Supplementary Figure S1e–h. Less iridescent color was observed in this secondary film. These results suggest that the film formation that occurs when the reaction mixture is kept stagnant may block or slow down the infusion of air into the reaction mixture. This phenomenon could account for the slower and incomplete progression of the reaction. Film formation was also observed for similar stagnant reactions involving DHI or dopamine, but these did not appear to show any significant iridescent color and did not prevent the reaction from proceeding to completion. For all other precursors, dark colors appeared at the interface of the air/reaction mixture when similar reactions were kept stagnant. The dark-colored materials slowly diffused into the lower parts of the reaction mixture and ultimately formed a homogenous-looking mixture.

### 2.2. Characterization of Purified Materials: vis/NIR and UV Absorbance

As documented in the literature, the absorbance spectra of MNs dispersed in water yield an exponentially declining profile with increasing wavelength, particularly in the vis/NIR region of the electromagnetic spectrum. Such absorbance spectra can be fitted with an exponential function as shown in Equation (1).

A = A_0_ e^−kλ^ or lnA = lnA_0_ − kλ (1)

In this equation, k is the decay constant of the exponential profile of absorbance (A) as a function of wavelength (λ). A_0_ is the absorbance of the sample when λ = 0. For quantitative evaluations, exponential regression analyses were performed for the absorbance readings between 500 and 800 nm to avoid the interference of components with strong absorbance bands in the upper UV region (300–400 nm) and to ensure that all absorbance readings were sufficiently above baseline. For light-colored materials, e.g., derived from epinephrine, caffeic acid, dihydrocaffeic acid, 3,4-dihydroxybenzoic acid or pyrogallol, regression analyses were performed between 500 and 700 nm.

Alternatively, some absorbance profiles in the vis/NIR region could be modeled with a reciprocal power function as shown in Equation (2).

(2) $$A=\frac{a}{\lambda^{z}}\;+\;b$$

**Figure 2:**
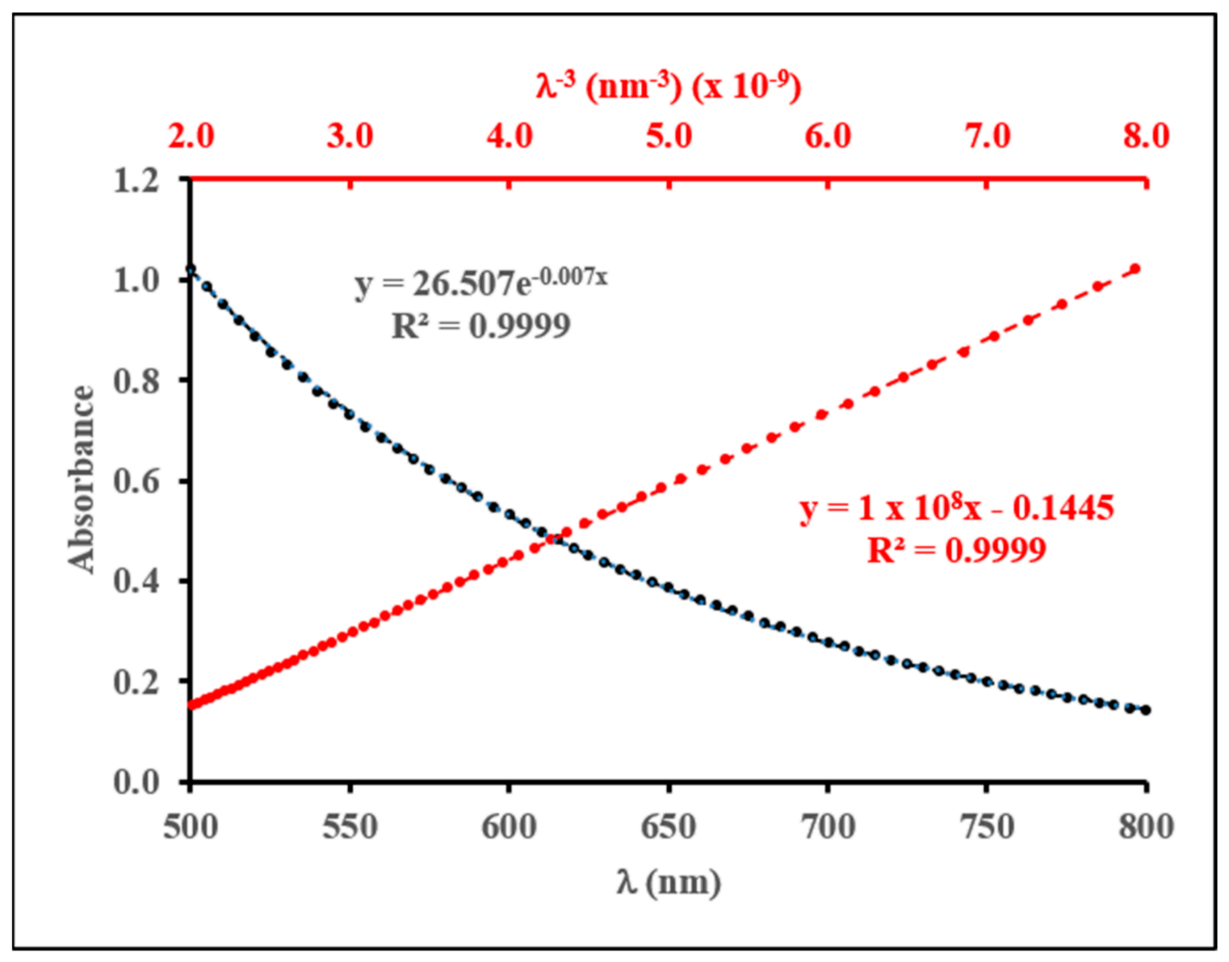
Comparison between the modeling of experimental absorbance readings obtained from a purified L-DOPA-based MN dispersed at 275 μg/mL with an exponential function (Equation (1); black)) or reciprocal power function (Equation (2); red)).

In this equation, A is the absorbance, z is a constant that can be obtained through an iteration process (the Solver add-in available for Microsoft Excel^®^ was used to obtain the value of z that provides the best linear fit through the experimental data as judged by the value of the correlation coefficient (r^2^)), and a and b are the values of the slope and intercept of the line relating A to 1/λ^z^. The application of Equations (1) and (2) is illustrated in Figure 2 for a dispersion containing purified L-DOPA-based MN.

To objectively quantify the absorbance intensity in the vis/NIR region of any given MN dispersion, Equations (1) and (2) can be integrated between 400 and 900 nm using the established values of A_0_ and k, or a, b and z respectively. Such integrations, shown in Equations (3) and (4), represent the area under the curve, termed AUC_vis/NIR_, and represent the total absorbance of the spectrum within the vis/NIR region of the electromagnetic spectrum.

(3) $${\mathrm{AUC}}_{\mathrm{vis}/\mathrm{NIR}}=\int_{400}^{900}A_{0}\times e^{-k\lambda }d\lambda =\frac{A_{0}}{-k}\times \left(e^{-k\;\times \;900}-e^{-k\;\times \;400}\right)$$

(4) $${\mathrm{AUC}}_{\mathrm{vis}/\mathrm{NIR}}=\int_{400}^{900}\left(\frac{a}{\lambda^{z}}+b\right)d\lambda =\frac{a}{-z+1}\times \left(900^{-z+1}-400^{-z+1}\right)+500\;\times \;b$$

The value of A_0_ in Equation (1) depends on the concentration of the sample, while the value of k is independent of the concentration and is to be considered a material-specific parameter. Similarly, the values of a and b in Equation (2) depend on the concentration of the sample, while the value of z is independent of the concentration and is to be considered a material-specific parameter. These facts are illustrated in Supplementary Figure S2a–d, for L-DOPA-based MN. Figure S2a shows absorbance profiles in the vis/NIR region of the same sample dispersed at varying concentrations. Figure S2b compares values of AUC_vis/NIR_, calculated using Equation (3) or (4), as a function of sample concentration. The results indicate that identical values for AUC_vis/NIR_ are obtained with both equations. Figure S2c shows the linear relationship between the value of A_0_ from Equation (1) and sample concentration, and includes the average value (±standard deviation; n = 7) of k from Equation (1). Figure S2d shows the linear relationship between the values of a and b from Equation (2) and sample concentration, and includes the average value (± standard deviation; n = 7) of z from Equation (2).

The absorbance in the UV region by MNs cannot be approached with a simple mathematical equation due to the presence of specific absorbance bands within that region. To obtain an objective estimate of the total absorption in the UV region of the electromagnetic spectrum (between 230 and 400 nm), the AUC in that region, termed AUC_UV_, was calculated as shown in Equation (5).

(5) AUCUV=∑230395Aλ+Aλ+∆λ2 × ∆λ with ∆λ=5 nm

Supplementary Figure S2e shows absorbance profiles in the UV region of the same samples shown in Supplementary Figure S2a. Supplementary Figure S2f shows the linear relationship between the value of AUC_UV_ and sample concentration. The parameters AUC_vis/NIR_ and AUC_UV_ depend on the concentration (C) of the MN dispersion. However, the slope of the relationship between any AUC value and C, as shown in Supplementary Figure S2b–f, represents the values of AUC_vis/NIR_/C or AUC_UV_/C. These values are to be considered material-specific parameters and can be used as objective parameters to define the MN’s capacity to absorb light in the vis/NIR or UV region of the electromagnetic spectrum. Supplementary Table S1 lists the values of AUC_vis/NIR_/C and AUC_UV_/C obtained for the various materials synthesized. In addition, Supplementary Table S2 provides photographs of melanin materials synthesized form pyrogallol, dopamine or DHI dispersed at varying concentrations to showcase the range of appearances that can be obtained. A parameter like AUC_vis/NIR_ provides an objective, quantitative evaluation of the intensity of the appearance (“color”) of a particular MN dispersion. However, it does not provide an objective, qualitative evaluation of the nature of the appearance of the MN material: dark-colored vs. light-colored. To objectively evaluate the nature of the appearance of MN dispersions, the ratio of absorbance at 650 nm over absorbance at 500 nm (A_650_/A_500_) was evaluated as a function of the value of k from Equation (1) as has been discussed elsewhere [10]. Although these two wavelengths are arbitrarily chosen, it follows precedent as a suggested way to differentiate dark-colored EuMN from light-colored PhMN [37,38,39]. Given the exponential relationship between A and λ (see Equation (1)), an exponential relationship exists between k and A_650_/A_500_ as outlined in Equation (6).

(6) $$\frac{A_{650}}{A_{500}}\;=\;\frac{A_{0}\;\times \;e^{-k\;\times \;650}}{A_{0}\;\times \;e^{-k\;\times \;500}}\;=\;\frac{e^{-k\;\times \;650}}{e^{-k\;\times \;500}}\;=\;e^{-k\;\times \;(650-500)}\;=\;e^{-k\;\times \;150}$$

**Figure 3:**
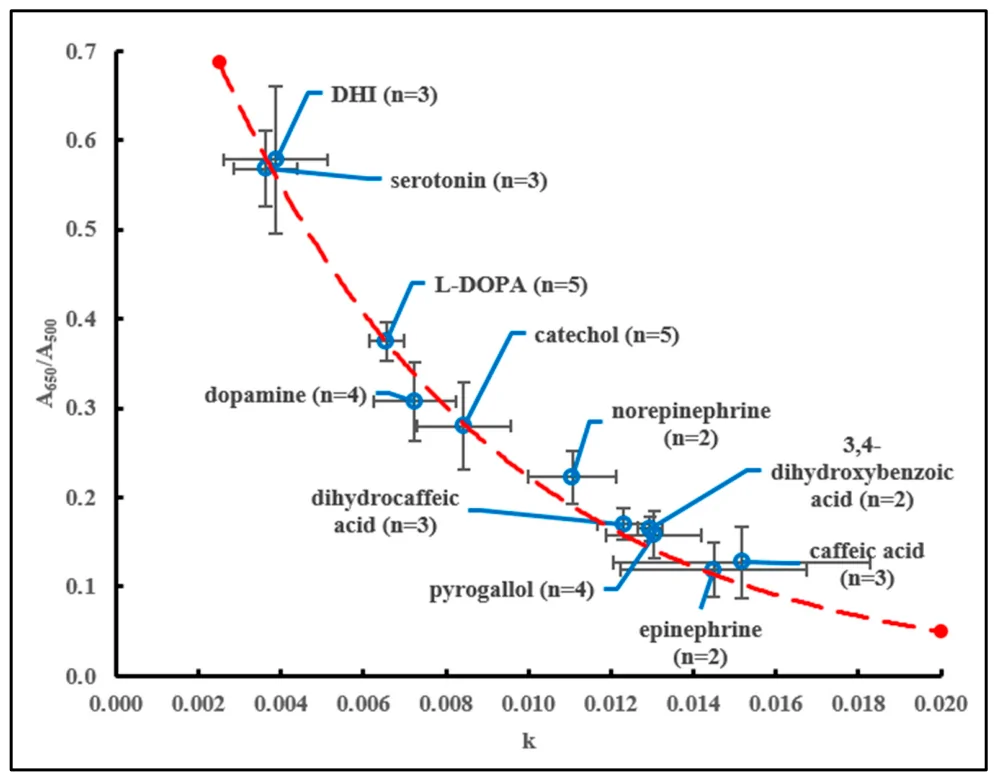
Plot of average (±standard deviation) value of k as function of average (±standard deviation) value of A_650_/A_500_ according to Equation (6) for purified materials synthesized from varying precursors and evaluated at varying concentrations as outlined in Section 4.4. The dashed red line represents the theoretical relationship between k and A_650_/A_500_ according to Equation (6).

Higher values of k and lower values of A_650_/A_500_ typically indicate light-colored (orange to yellow) materials. Lower values of k and higher values of A_650_/A_500_ typically indicate dark-colored (brown to black) materials. Figure 3 shows a plot of the average value of k (±standard deviation) vs. the average value of A_650_/A_500_ (±standard deviation) for the purified materials prepared from varying precursors as listed in Supplementary Table S1. The experimental data points are plotted in relationship to the theoretical line according to Equation (6). Reactions involving serotonin consistently resulted in precipitated materials. After washing with water, the dark-colored material could be dispersed in 1 N HCl and absorbance spectra could be recorded and modeled according to Equation (1).

All the experimental data shown in Figure 3 fall on, or are close to, the theoretical line. This suggests that, despite the differences in chemical structures of the precursors, the materials derived all share the same property of exhibiting an exponential relationship between the absorbance and the wavelength in the vis/NIR region.

### 2.3. Characterization of Purified Materials: FT-IR Spectroscopy

**Figure 4:**
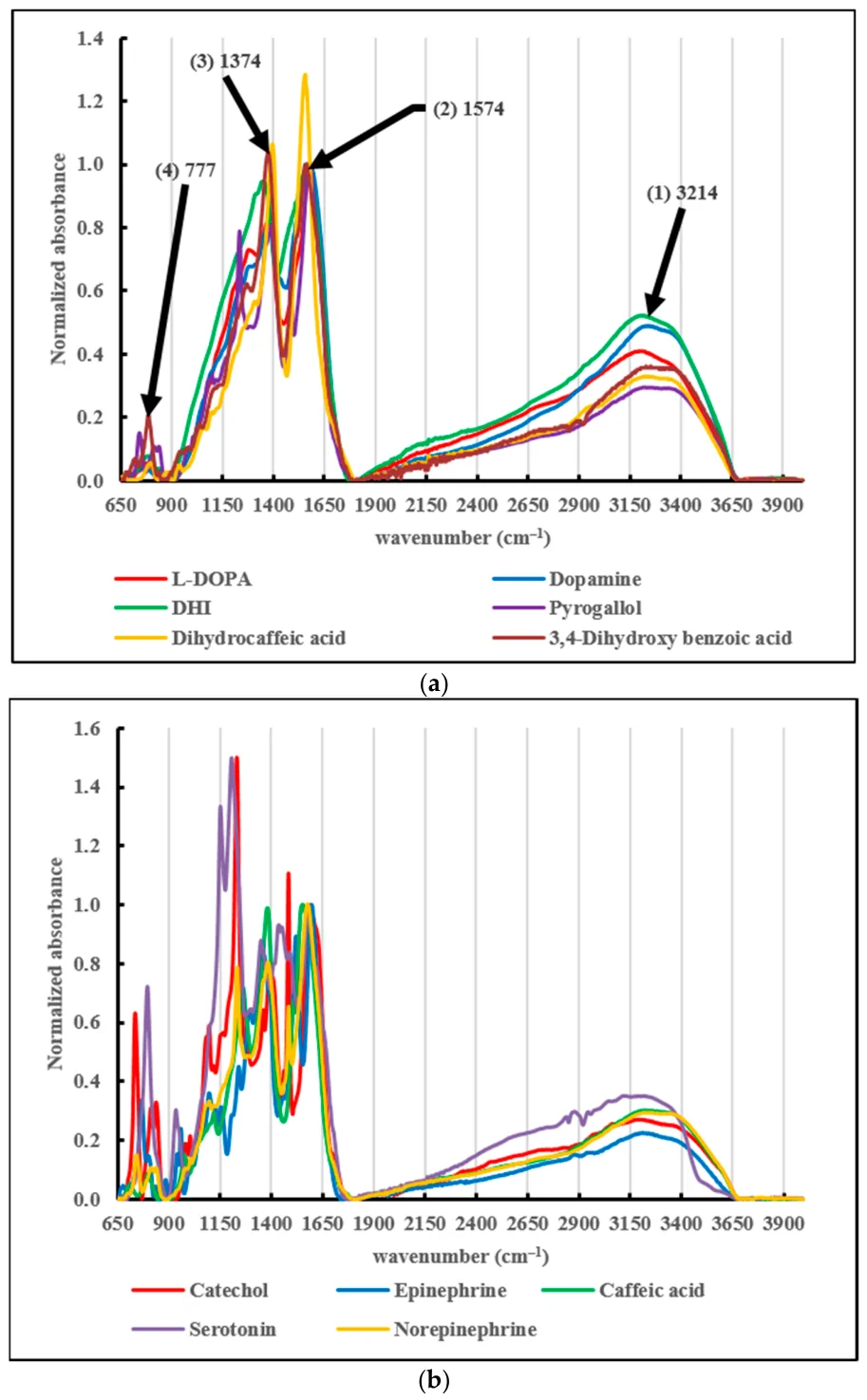
(**a**) Normalized FT-IR spectra of select MNs derived from L-DOPA, dopamine, DHI, pyrogallol, dihydrocaffeic acid, and 3,4-dihydroxybenzoic acid. (**b**) Normalized FT-IR spectra of select MNs derived from catechol, epinephrine, caffeic acid, serotonin and norepinephrine. The numbered arrows in panel A indicate common signals in all spectra and are discussed in the text.

Purified MNs were scanned using FT-IR spectroscopy. Raw data of these scans exhibited a distinct signal around 1580 cm^−1^ indicative of the ring mode of aromatic rings [40]. Thus, all spectra were normalized for their signal associated with this ring mode. Figure 4 shows an overlay of select FT-IR profiles obtained from MNs derived from different precursors following the dialysis of the crude reaction mixture. For clarity purposes the FT-IR profiles were divided over two different panels.

**Figure 5:**
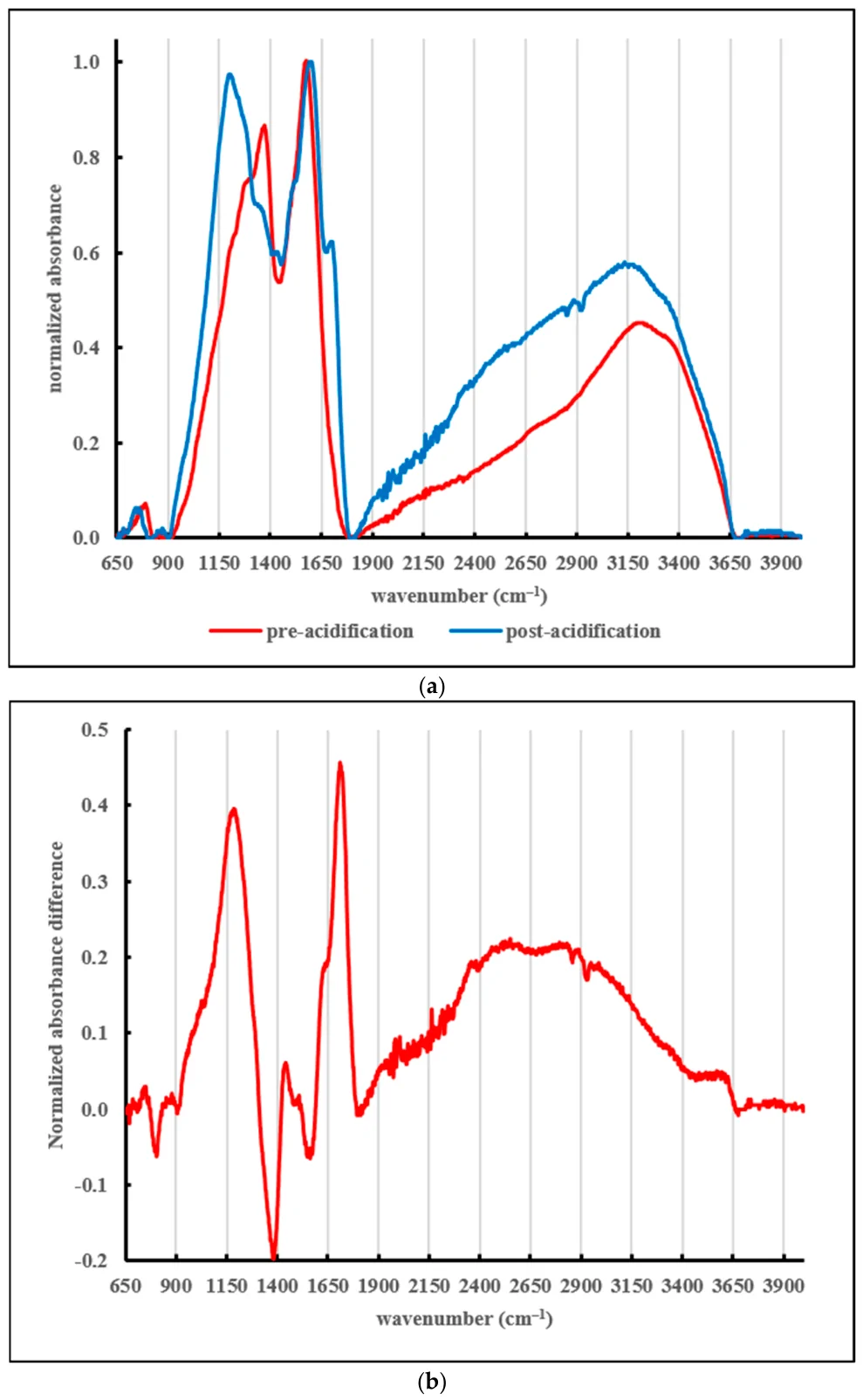
(**a**) Normalized FT-IR spectra of L-DOPA-based MN pre- and post-acidification. (**b**) Absorbance difference following subtraction of normalized FT-IR spectrum of L-DOPA-based MN pre-acidification, from the normalized FT-IR spectrum of the same material post-acidification.

The FT-IR profiles shown in Figure 4a exhibit broad signals, while the FT-IR profiles shown in Figure 4b exhibit an array of sharper signals. For each type of material, the characteristic peak maxima in their FT-IR spectra are listed in Supplementary Table S1. Despite the chemical differences of the precursors used, all FT-IR profiles share signals in common areas of the spectrum. The numbered arrows shown in Figure 4a indicate peak maxima observed in all FT-IR profiles, independent of the precursor used in the synthesis of the MN. A total of 35 materials, derived from various precursors, were evaluated using FT-IR spectroscopy. The average (±standard deviation) of the peak maxima 1 through 4 shown in Figure 4a were observed at: (1) 3214 ± 19 cm^−1^, (2) 1574 ± 13 cm^−1^, (3) 1374 ± 12 cm^−1^ and (4) 777 ± 21 cm^−1^. These signals could be assigned to (1) O-H, and, if applicable, N-H stretch, (2) ring mode of aromatic ring, (3) O-H bend and (4) out-of-plane C-H bend of aromatic ring [40,41,42]. As will be discussed later in this report, for MNs purified through dialysis, carboxylic acid functional groups may exist in carboxylate form. Thus, the signals associated with the asymmetric and symmetric C-O stretch of carboxylates, which typically present as a pair of signals between 1540 and 1650 cm^−1^ and between 1360 and 1450 cm^−1^ [43], overlap with some of the features discussed above. None of the profiles in Figure 4a,b show a distinct signal that would indicate the presence of a carbonyl group [44]. Such a carbonyl group could be associated with the presence of a carboxylic acid, aldehyde or ketone functional group. Even the FT-IR spectra of MNs made from L-DOPA, caffeic acid, dihydrocaffeic acid or 3,4-dihydroxybenzoic acid do not show a signal associated with a carbonyl. However, given the alkaline conditions of the synthesis process, and dialysis as the purification process, it can be assumed that any carboxylic acid functional group present existed in its carboxylate state. To evaluate this possibility, select purified MNs were acidified with HCl and purified anew as outlined in Section 4.4. Acidified MNs were scanned using FT-IR spectroscopy. Figure 5a shows a comparison between the normalized FT-IR spectra of a L-DOPA-based MN pre- and post-acidification. Figure 5b shows the normalized FT-IR spectrum of the MN pre-acidification, subtracted from the FT-IR spectrum of the MN post-acidification. Supplementary Figure S3a–f  show similar profiles obtained for MNs synthesized from other precursors.

**Figure 6:**
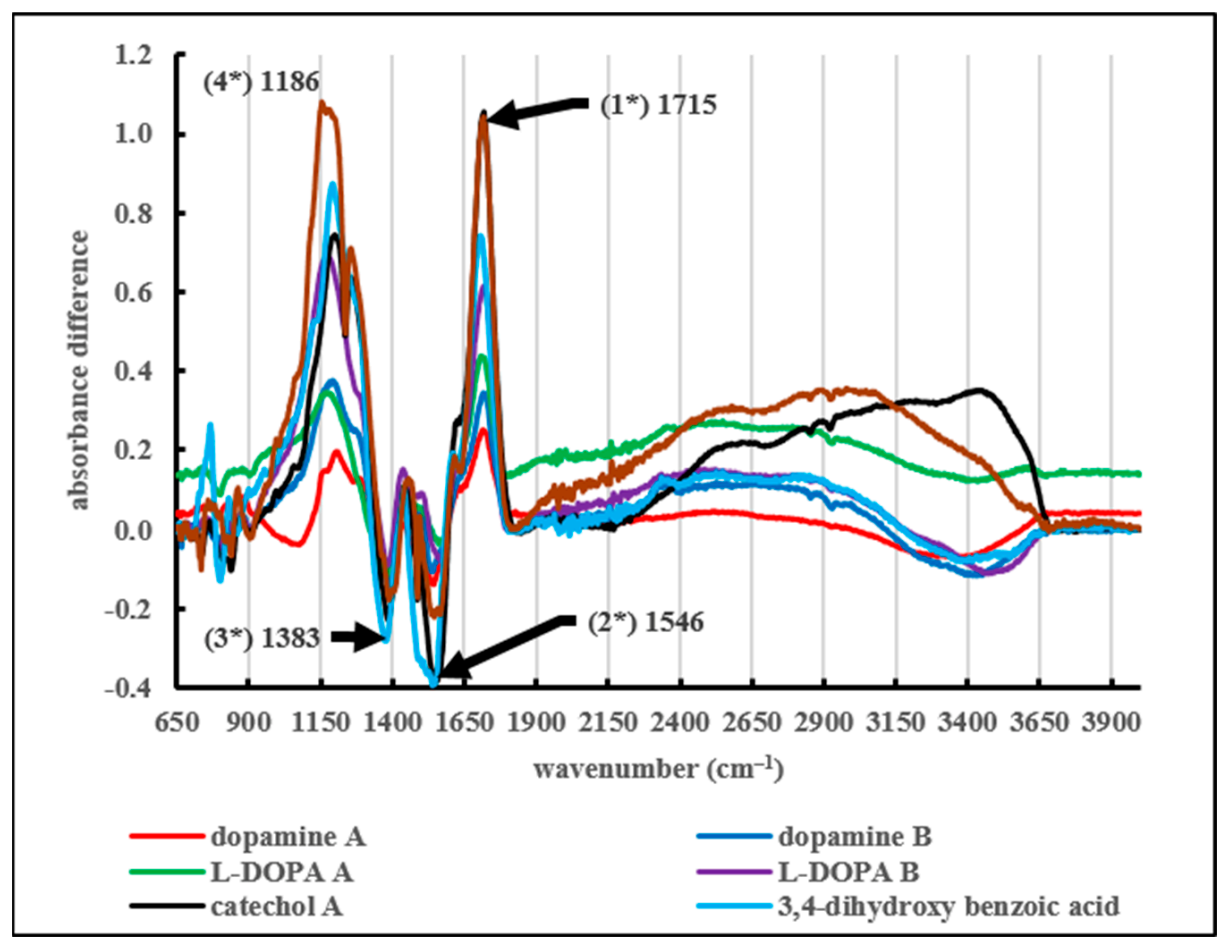
Absorbance difference of normalized FT-IR spectra, obtained by subtracting the FT-IR spectrum pre-acidification from the FT-IR spectra post-acidification, of MNs derived from various precursors. Two batches (designated as A or B) of materials synthesized from dopamine, L-DOPA or catechol were evaluated. The numbered arrows indicate common features in all the subtracted, normalized FT-IR absorbance profiles and are discussed in the text. “*” was added to the wavenumbers to distinguish them from the wavenumbers highlighted in Figure 4, panel (a).

All FT-IR spectra of acidified MNs exhibited a peak or shoulder in the low 1700 cm^−1^ region of the spectrum as shown in Figure 5a, and Supplementary Figure S3a,c,e. The subtracted FT-IR profiles shown in Figure 5b, and Supplementary Figure S3b,d,f, show a positive peak in the low 1700 cm^−1^ region, and other common negative or positive features. Figure 6 presents an overlay of all (n = 7) such subtracted absorbance profiles obtained from various MNs.

As indicated in Figure 6, the subtracted, normalized FT-IR spectra show four common signals independent of the type of precursor used in the synthesis of the MN. The average (±standard deviation; n = 7) position of these peaks, positive or negative, are: (1*) 1715 ± 5 cm^−1^, (2*) 1546 ± 9 cm^−1^, (3*) 1383 ± 5 cm^−1^ and (4*) 1186 ± 17 cm^−1^. The positive signal at 1715 cm^−1^ can be assigned to the C=O stretch of a carboxylic acid functional group that emerged post-acidification [45]. The negative signals at 1546 and 1383 cm^−1^ can be assigned to the disappearance of the asymmetric and symmetric C-O stretch of a carboxylate functional group post-acidification [43]. The positive signal at 1186 cm^−1^ can be assigned to the C-O stretch of a carboxylic acid functional group, possibly overlapping with the C-O stretch of phenols [45,46]. Although acidification of the MN would render any amine functional group into its protonated form, the N-H stretching or bending vibrations may be too weak to be readily distinguishable in the FT-IR spectra of the complex MN [47]. In addition to the observations illustrated in Figure 6, it appeared that the signal associated with the aromatic ring mode shifted from an average of 1583 ± 6 cm^−1^ (n = 35; see Figure 4) pre-acidification, to an average value of 1601 ± 5 cm^−1^ (n = 7) in the FT-IR spectra of the materials post-acidification.

### 2.4. Precipitation Experiments Using CaCl_2_

As outlined in Section 4.5, select MNs were dispersed in water and mixed with Ca^2+^ at varying concentrations. After overnight standing, the absorbance of the supernatant in the UV to NIR region of the spectrum, termed AUC_total_, was calculated according to Equation (7).

(7) AUCtotal=∑230895Aλ +Aλ+∆λ2×∆λ with ∆λ=5nm

**Figure 7:**
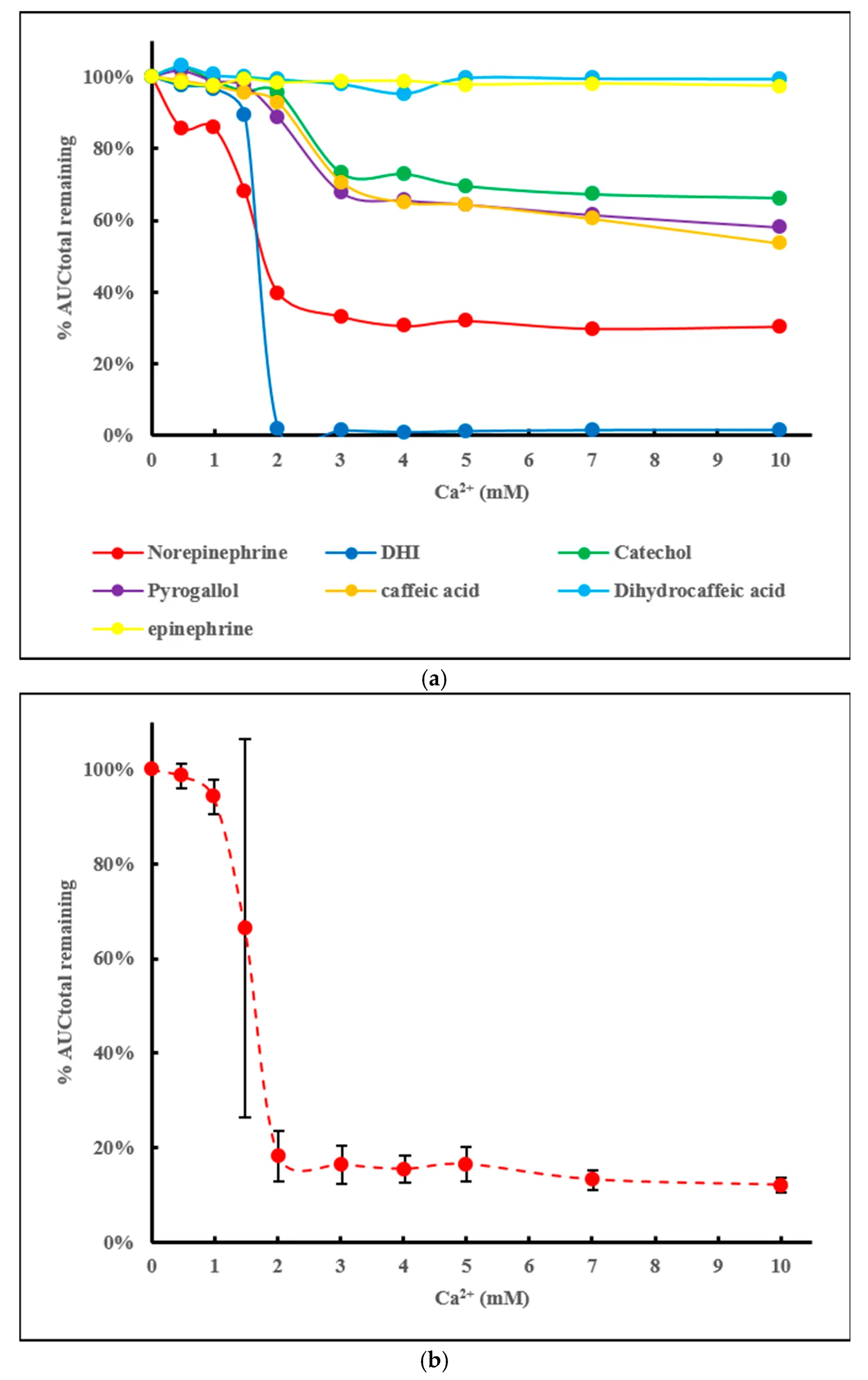

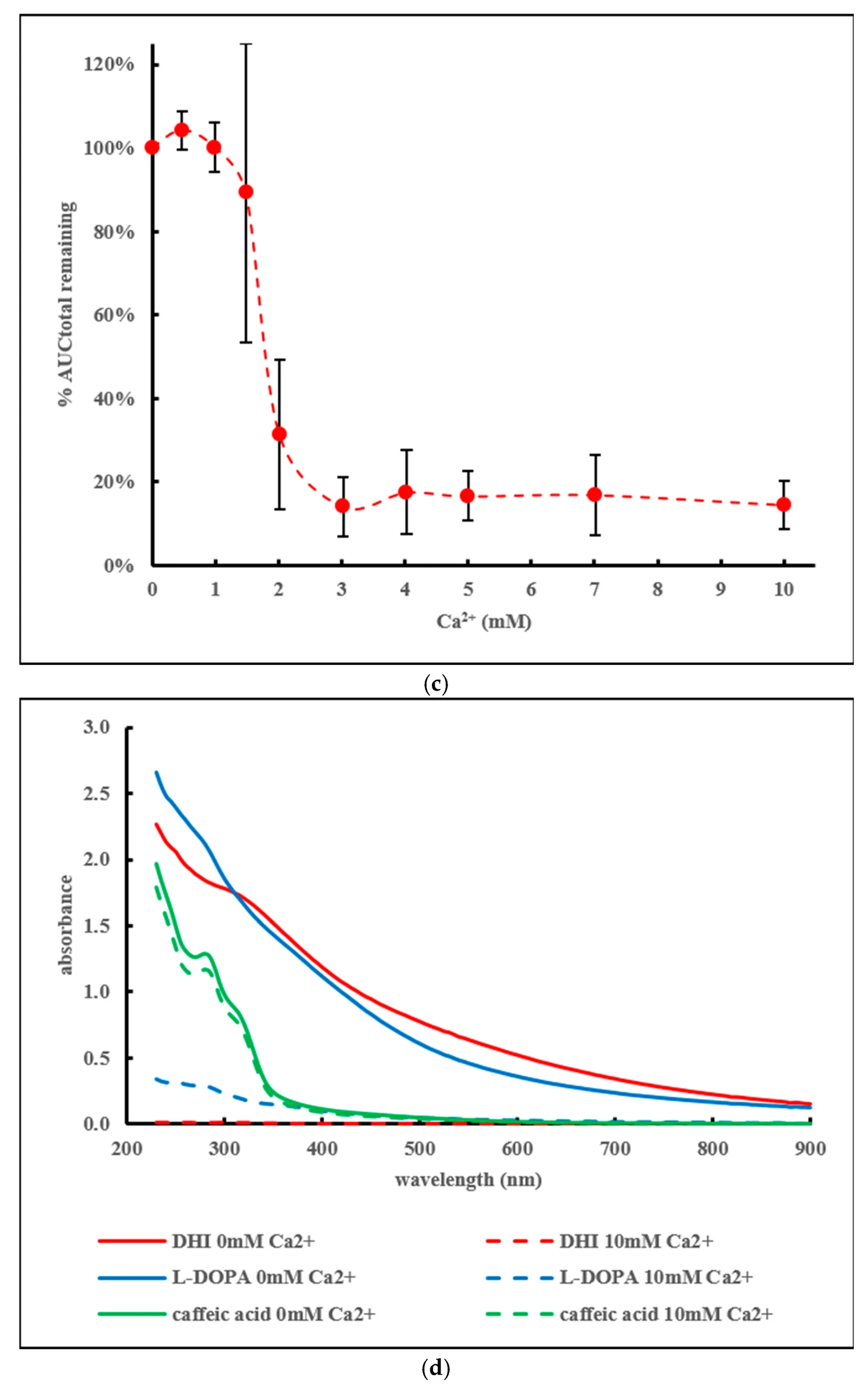
(**a**) Relative AUC_total_, as calculated using Equation (7), of the supernatant of dispersions containing MN, synthesized from various precursors, in the presence of increasing concentrations of Ca^2+^. (**b**) Average (±standard deviation, n = 3) relative AUC_total_, as calculated using Equation (7), of the supernatant of dispersions of three MN batches synthesized from dopamine, in the presence of increasing concentrations of Ca^2+^. (**c**) Average (±standard deviation, n = 3) relative AUC_total_, as calculated using Equation (7), of the supernatant of dispersions of three MN batches synthesized from L-DOPA, in the presence of increasing concentrations of Ca^2+^. (**d**) Absorbance profiles of the supernatant of mixtures containing MN synthesized from DHI, L-DOPA or caffeic acid and mixed with 0 or 10 mM Ca^2+^.

For each mixture the % AUC_total_ remaining in the supernatant, compared to the mixture containing no Ca^2+^, was calculated and plotted as a function of Ca^2+^ concentration. Figure 7a presents typical results obtained for MNs synthesized from different precursors. For the precursors listed in Figure 7a, two different batches of MN were tested, but for clarity purposes the results of only one batch of each are shown as the results were consistent amongst the duplicate batches of MNs. Figure 7b,c, present average results obtained from experiments involving three different dopamine-based (Figure 7b) or three different L-DOPA-based (Figure 7c) batches of MN. Figure 7d illustrates the absorbance profiles of MNs synthesized from DHI, L-DOPA or caffeic acid mixed with 0 or 10 mM Ca^2+^.

For all MNs tested, with the notable exceptions of dihydrocaffeic acid- and epinephrine-based MNs, the addition of Ca^2+^ resulted in the precipitation of dark-colored substances. However, this precipitation occurred in a non-linear fashion with increasing concentration of Ca^2+^. A sharp decline in the values of AUC_total_ was typically observed within a narrow range of Ca^2+^ concentration, particularly in the cases of DHI (see Figure 7a), dopamine (see Figure 7b) and L-DOPA (see Figure 7c). The high variations observed for the results with dopamine and L-DOPA for Ca^2+^ concentrations around 1.5 mM is a reflection that this level of Ca^2+^ was the inflection point at which the MN dispersion was physically stable or unstable. Figure 7d illustrates the fact that, when maximum precipitation by Ca^2+^ was achieved, some residual material remains present in the supernatant as studied previously [11].

## 3. Discussion

The observations shown in Supplementary Figure S1 reflect the capacity of MN synthesized from serotonin to spontaneously generate macroscopic structures with iridescent properties. These observations, and to a lesser extent with dopamine or DHI, reflect the capacity of MNs to generate structural colors as discussed elsewhere [6,33,48]. Serotonin-based MN kept in an alkaline environment never yielded a physically stable dispersion. However, it can be dispersed in an acidic environment. When dispersed in an acidic environment, it appears akin to EuMN, and the FT-IR spectrum of serotonin-based MN has the same key features as the other MNs we evaluated (see Figure 4b). The physical instability of serotonin-based MN would lend this type of material suitable for coating applications as is extensively studied for dopamine- or norepinephrine-based MN. In a broader sense, the hydroxyindole class of compounds could serve as suitable precursors to synthesize MN-like materials and to be evaluated for applications similar to dopamine- or norepinephrine-based materials.

The interaction between electromagnetic radiation and MN is extensively discussed by Xie et al. [6]. Despite the complexities of the interactions between electromagnetic radiation and MN, the outcome is consistently simple: a smooth, exponentially declining absorbance with increasing wavelength. Although the quantitative aspects of this property may differ depending on the precursor used or the reaction conditions, the qualitative aspects are consistent (there is never a “bump” in the absorbance profile). Any model invoked to explain the appearance of MN should explain this consistency independent of the precursor employed. As mentioned in the introduction, the chemical diversity model has been suggested to explain the absorbance profile of MN materials. This would suggest that every time MN is synthesized, a collection of compounds is generated in the exact ratio as to generate the smooth exponential absorbance profile, even when chemically different precursors are employed. In addition, this model would suggest that, e.g., L-DOPA-based MN, could be fractionated into a set of compounds that, when displayed side by side, would represent the colors of the rainbow. There is very little evidence of this to be found in the literature. The in vitro synthesis of MN does lead to a set of chemically diverse entities, as evidenced by the complexity of the FT-IR spectrum of the MN, but it is unlikely that their individual absorbance spectra could explain the overall absorbance spectrum of the MN. The absorbance profile exhibited by MN is readily modeled with an exponential equation (see Equation (1) and Figure 2). However, this report shows that an alternative mathematical model can be used to approach the same experimental absorbance profiles (see Equation (2) and Figure 2). The results presented in Figure 2 and Supplementary Figure S2 illustrate that both Equations (1) and (2) are suitable to objectively evaluate and quantify the vis/NIR absorbance properties of MNs, particularly EuMN-like materials. The use of the reciprocal power function, and the estimation of the corresponding material-specific parameter z from Equation (2), is novel. Equation (2) provides an alternative foundation to develop theoretical models to describe the interaction between MN and electromagnetic radiation. In this context it can be pointed out that for some interactions between electromagnetic radiation and material, e.g., Rayleigh scattering, a mathematical model involving a reciprocal power factor of λ is applied. As will be discussed later, MNs present themselves as physical, colloidal entities. Thus, it is possible that a combination of chemical and physical features determines the interaction with electromagnetic radiation and that a single model cannot explain the absorbance spectrum of MN. However, a unifying concept has been proposed by Madhu et al. through spectroscopic studies of L-DOPA-based MN and other dark-colored natural organic materials (NOM) [49]. Their studies suggested that the chromophores responsible for the appearance of MN and NOM consist of stacks, a few nanometers in size, of oligomeric species aggregated through π-stacking. Based upon the suggestions made by Madhu et al. [49], one can assume that when synthesizing MN from different precursors, chemically different oligomers are generated that, due to differences in chemistry or charges, will lead to differences in nanoscale stacking, leading to differences in appearances. When considering MN materials as particulate or colloidal entities created by the stacking of oligomeric species, then four dimensions need to be considered: height, length, width, and the dynamics (time dimension) of the particle. The non-covalently bonded entities creating the MN particle could exhibit lateral, rotational or vertical motions relative to each other within the MN particle when dispersed in water at room temperature. The chemical diversity present in the MN particle could affect all four of these dimensions, which then may impact the interaction of the MN particle with electromagnetic radiation.

Both parameters k and z, from Equations (1) and (2) respectively, are material-specific parameters related to the overall appearance of the MN material: darker, EuMN-like materials are characterized by lower values of k or z, while lighter, PhMN-like materials are characterized by higher values of k or z. In addition, through mathematical simulations it was observed that a linear relationship exists between the values of k and z. Thus, for every value of k, there is a value of z for which Equation (8) holds.

(8) $$A_{0}\;e^{-\mathrm{k\lambda}}\approx \;\frac{a}{\lambda^{z}}+b$$

Modeling experimental data according to Equation (2) allows for an estimate of the wavelength at which the absorbance is 0, termed λ_0_ as shown in Equation (9).

(9) $$\lambda_{o}=\sqrt[{z}]{-\frac{a}{b}}$$

Knowledge of λ_0_ allows for a calculation of the energy level at which the absorbance is 0, termed E_0_, via E_0_ (in eV) = 1239.8419/λ_0_. According to the Tauc equation, E_0_ represents an estimate of the band gap width of a material as it applies to semiconductor materials [50]. For the results obtained for the L-DOPA-based MN shown in Figure 2, E_0_ can be estimated as 1.24 eV which is in line with reported estimates [51,52,53,54]. Parameters like k or z are related to the type of color (dark or light). The dotted red line shown in Figure 3 correlates with the range of colors that can be generated depending on the precursor used: from black (upper left corner of red dotted line in Figure 3) to yellow (lower right corner of red dotted line in Figure 3). The pattern of results shown in Figure 3 suggests that precursors that have indole features in their chemical structures (serotonin, DHI) or could yield indole features through intramolecular substitution reaction by the primary amine (L-DOPA, dopamine, norepinephrine), yield darker colored MNs. Precursors that cannot yield indole features (pyrogallol, caffeic acid, dihydrocaffeic acid, epinephrine, 3,4-dihydroxybenzoic acid) yield lighter colored MNs. A notable exception is catechol which yields dark-colored MN on par with L-DOPA or dopamine. Epinephrine yields light-colored MN presumably because its secondary amine functionality is less suited for intramolecular substitution to yield an indole chemical feature. Parameters like AUC_vis_NIR_/C or AUC_UV_/C represent the intensity of absorbance (intensity of color) that can be achieved by any given MN and can be considered the equivalent of an absorption coefficient. Values of these parameters are listed in Supplementary Table S1. Supplementary Table S2 provides photographs of melanin materials synthesized from pyrogallol, dopamine or DHI, dispersed at varying concentrations. These photographs illustrate the range of appearances that can be achieved depending on the precursor and concentration employed. Objective, numerical values like AUC_vis_NIR_/C or AUC_UV_/C can provide guidance for developing tone-matching cosmetic products or selecting agents for photothermal therapy [54,55,56].

Due to its insolubility in water or organic solvent, the full chemical characterization of MN is still incomplete. FT-IR spectroscopy is commonly used although it provides only limited chemical information on the structure of MN. An overview of typical peak signals in FT-IR spectra of natural or synthetic MN is presented by Perna et al. [57]. The FT-IR analyses we performed on select MNs provide various observations. The spectra of the MNs shown in Figure 4 have similar features despite the diversity in the chemistry of the precursors involved. The FT-IR spectra of the MNs shown in Figure 4b exhibited sharper signals compared to the spectra shown in Figure 4a, but the peak maxima are within the same regions. Typical FT-IR peak values observed in the MNs synthesized for this report are listed in Supplementary Table S1. By comparing and subtracting the FT-IR spectra of MN before and after acidification, one can observe that many MNs contain carboxylic acid/carboxylate functionalities (see Figure 5 and Figure 6). Even MNs synthesized from precursors without carboxylic acids, e.g., dopamine or catechol, exhibited carboxylic acid/carboxylate features. This suggests that oxidative aromatic ring cleavage occurred during the synthesis reaction, a possibility that has been discussed elsewhere [58]. However, computational studies on MN structures often do not include building block units containing cleaved aromatic features [21,59,60,61]. Our results indicate that dialysis yields materials with carboxylate functionalities, rendering them anionic in nature. This is in line with earlier observations on a different set of MNs [25]. Thus, the purification process employed impacts the surface charge of MN which will impact their physical and chemical properties, and their potential applications.

The anionic nature of MNs allows for their interaction with multivalent cations as detailed here with Ca^2+^ and in a previous report with La^3+^ [11]. Figure 7a–c illustrate that the physical stability of MN in the presence Ca^2+^ is an “all or nothing phenomenon”: precipitation occurs only above a critical Ca^2+^ concentration. These results emphasize that synthetic MN presents itself as physical, colloidal particles as discussed in another report [11]. The results presented in Figure 7a–c suggest that the darker the MN, the greater the impact on the MN’s physical stability by the addition of Ca^2+^. In addition, the results shown in Figure 7d reinforce the notion that synthetic MN consists of at least two fractions: a dark-colored and a light-colored fraction [11]. The relative ratio of these two fractions within the MN depends on the precursor used for its synthesis as reflected in the % AUC_total_ remaining at the highest concentration of Ca^2+^ tested (see Figure 7). For DHI-based MN, nearly 100% of the AUC_total_ was lost upon precipitation with 10 mM Ca^2+^, while at the other extreme only 1 to 3% of AUC_total_ was lost in the cases of epinephrine- and dihydrocaffeic acid-based MN. Given the extensive studies on the properties and applications of MN-based coatings, the ability to control the physical instability of MN materials, e.g., through the addition of multivalent cations, is an important application of the reported observations.

## 4. Materials and Methods

### 4.1. Materials

Dopamine.HCl (Thermo Scientific, Waltham, MA, USA), L-DOPA (MP Biomedicals, Santa Ana, CA, USA), epinephrine.HCl (Sigma, St Louis, MO, USA), norepinephrine.HCl (Sigma, St Louis, MO, USA), catechol (Acros Organics, Geel, Belgium), pyrogallol (Sigma, St Louis, MO, USA), caffeic acid (Sigma, St Louis, MO, USA), dihydrocaffeic acid (Thermo Scientific, Waltham, MA, USA), 3,4-dihydroxybenzoic acid (Aldrich, St Louis, MO, USA), serotonin.HCl (Thermo Scientific, Waltham, MA, USA), and 5,6-dihydroxyindole (DHI; Thermo Scientific, Waltham, MA, USA) were all purchased through Fisher Scientific (Waltham, MA, USA) but originated from different suppliers as indicated.

### 4.2. UV and Vis/NIR Spectroscopy

UV and vis/NIR spectroscopic measurements were made in wells of a 96-well, UV-transparent microplate using a SynergyHT microplate reader from Biotek (Winooski, VT, USA). Spectra were recorded with 5 nm intervals.

### 4.3. FT-IR Spectroscopy

FT-IR analyses were performed using the Spectrum Two FT-IR spectrometer from PerkinElmer (Waltham, MA, USA). Scans were made using the universal ATR accessory between 650 and 4000 cm^−1^ with a resolution of 4 cm^−1^ and using the OptKBr beam splitter and LiTaO_3_ detector. For each sample 24 scans were accumulated. FT-IR spectra were normalized for their signal at 1580 cm^−1^ as this signal, associated with the ring mode of aromatic entities, is common in the spectra of all the materials.

### 4.4. Synthesis and Evaluation of Purified Materials

Reactions were set up by dispersing between 200 and 300 mg precursor in 100 mL 25 mM Na_2_CO_3_. Crude reaction mixtures were purified through dialysis followed by freeze-drying. Depending on the availability of the precursor molecules, between 2 and 5 different batches were made from each precursor as shown in Supplementary Table S1. Dried materials were scanned using FT-IR spectroscopy. In addition, purified materials were dispersed in distilled water and scanned for their absorbance in the UV to NIR range at varying concentrations. Select purified materials were dispersed in water to a concentration of 6 mg/mL. This dispersion was acidified using HCl to a final concentration of 0.1 N. If precipitation occurred, precipitates were centrifuged, washed with water and freeze-dried. If no precipitation occurred, acidified dispersions were dialyzed and freeze-dried. FT-IR spectra were recorded of all acidified materials. Separately, 100 mg serotonin.HCl was dissolved in 40 mL 50 mM Na_2_CO_3_. This mixture was kept stagnant at room temperature for up to ten days. The reaction was monitored visually, and photographs were taken on various occasions. Other reactions involving serotonin were set up by dissolving 100 mg serotonin.HCL in 35 mL 25 mM Na_2_CO_3_. These mixtures were kept in 50 mL polypropylene centrifuge tubes on a rotating wheel to ensure proper mixing (polypropylene reaction containers were chosen to avoid irreversible staining of glassware by the reaction products). At the end of the reaction, the reaction mixtures were centrifuged, and precipitates were washed with water, freeze-dried and scanned with FT-IR spectroscopy. Dried material was dispersed in 0.1 N HCl to a concentration between 1 and 2 mg/mL and scanned for its absorbance in the UV to NIR range at varying concentrations.

### 4.5. Precipitation Studies Using CaCl_2_

Purified MNs were dispersed in distilled water at a concentration of 1 mg/mL. A total of 450 μL of dispersion was mixed with 50 μL CaCl_2_ dilutions such that the Ca^2+^ concentration varied between 0 and 10 mM. After overnight standing, mixtures were centrifuged and supernatants, diluted 8-fold, were scanned for their absorbance in the UV to NIR range.

### 4.6. Dialysis and Freeze-Drying

Dialysis was performed using Spectrum Spectra/Por RC dialysis membranes with molecular-weight-cut-off (MWCO) of 3.5 kDa obtained from Fisher Scientific (Suwanee, GA, USA). Samples were freeze-dried using a Labconco FreeZone Plus 4.5 L benchtop freeze-dry system obtained from Fisher Scientific (Suwanee, GA, USA).

## 5. Conclusions

This report details an evaluation of spectroscopic (absorbance, FT-IR) features present in MN materials synthesized from a diverse range of precursors. Despite the quantitative differences, all MNs studied had qualitatively similar features, independent of the differences in chemistry between the precursors employed. Mathematical models allowing for an objective evaluation of the quantitative aspects of MN’s absorbance features are presented. One such model provides the possibility to estimate the band gap width of MN. In addition to highlighting common features, FT-IR spectroscopy was employed to evaluate the presence of carboxylic acid or carboxylate functional groups and provide evidence of possible oxidative cleavage of aromatic rings during the synthesis of MN. Through precipitation experiments using Ca^2+^, the physical (in)stability of colloidal MN dispersions was evaluated and compared. It was observed that colloidal dispersions of dark-colored MN exhibited more pronounced physical instability compared to light-colored MN.

## Data Availability

The original contributions presented in the study are included in the article/Supplementary Materials. Further inquiries can be directed to the corresponding author.

## Supplementary Materials

The following supporting information can be downloaded at https://www.mdpi.com/article/10.3390/molecules31203687/s1, Supplementary Table S1: Overview of melanins synthesized from varying precursors and their characteristics as outlined in the main text; Supplementary Table S2: pictures of melanin samples synthesized from pyrogallol, dopamine or DHI dispersed at varying concentrations (Photographs by K.V.); Supplementary Figure S1: Top row: photographs of the top view of a stagnant reaction mixture containing serotonin (see Section 2.1). (a) Perpendicular view after 24 h of reaction, (b) angled view after 24 h of reaction, (c) angled view after 26 hours of reaction and (d) angled view after 7 days of reaction. Bottom row: photographs of secondary film formation (see Section 2.1). (e) angled view after 2 days, (f) angled view after three days, (g) angled view after six days and (h) angled view after seven days of additional reaction.(Photographs by K.V.); Supplementary Figure S2: (a) Absorbance profiles in vis/NIR region of L-DOPA based MN dispersed in water at varying concentrations. (b) Comparison of the values of AUCvis/NIR calculated using Equation (4) or Equation (5) as a function of sample concentration. (c) Linear relationship between the value of A0 from Equation (1) as a function of sample concentration and average value (± standard deviation; n = 7) of k from Equation (1). (d) Linear relationships between the values of a (red) or b (blue) from Equation (2) as a function of sample concentration and average value (± standard deviation; n = 7) of z from Equation (2). (e) UV absorbance profiles of L-DOPA based MN dispersed in water at varying concentrations. (f) Relationship between the values of AUCUV calculated using Equation (6) and sample concentration; Supplementary Figure S3: (a) Normalized FT-IR spectra of 3,4-dihydroxybenzoic acid-based MN pre- and post-acidification. (b) Absorbance difference following subtraction of normalized FT-IR spectrum of 3,4-dihydroxybenzoic acid-based MN pre-acidification, from the normalized FT-IR spectrum of the same material post-acidification. (c) Normalized FT-IR spectra of dopamine-based MN pre- and post-acidification. (d) Absorbance difference following subtraction of normalized FT-IR spectrum of dopamine-based MN pre-acidification, from the normalized FT-IR spectrum of the same material post-acidification. (e) Normalized FT-IR spectra of catechol-based MN pre- and post-acidification. (f) Absorbance difference following subtraction of normalized FT-IR spectrum of catechol-based MN pre-acidification, from the normalized FT-IR spectrum of the same material post-acidification.

## Abbreviations

The following abbreviations are used in this manuscript:

MN	melanin
EuMN	eumelanin
PhMN	pheomelanin
DHI	5,6-dihydroxyindole
UV	ultraviolet
Vis	visible
NIR	near infrared
FT-IR	Fourier Transform Infrared
A	absorbance
λ	wavelength
AUC	area under the curve
NOM	natural organic material
