# Supplementary material for: Spectroscopic Study of the Common Features of Melanins Synthesized from a Diverse Range of Precursors
Source: Molecules. 2026 Oct 15;31(20):3687. doi: 10.3390/molecules31203687 (PMC13707885; doi:10.3390/molecules31203687)
Supplement: Supplementary file 1 [file molecules-31-03687-s001.zip › molecules-4561024-supplementary.pdf]

Spectroscopic study of the common features of melanins synthesized from a diverse range of precursors

Koen Vercruysse

Supplemental Materials

**Supplementary Table S1: Overview of melanins synthesized from varying precursors and their characteristics as outlined in the main text.**				
**Precursor used to synthesize MN**	**AUC_{vis_NIR}/C (μg/mL)$^{-1}$**	**AUC_{UV}/C (μg/mL)$^{-1}$**	**k**	**Main FT-IR peaks (cm^{-1})**
L-DOPA (n = 5)	1.1 ± 0.16	2.2 ± 0.33	0.0066 ± 0.0004	3202 ± 6.1<br>1583 ± 3.3<br>1375 ± 0.55<br>785 ± 10
Dopamine (n = 4)	0.52 ± 0.044	1.5 ± 0.082	0.0072 ± 0.0004	3228 ± 12<br>1581 ± 1.3<br>1378 ± 4.7<br>779 ± 24
Catechol (n = 5)	0.25 ± 0.086	1.2 ± 0.25	0.0084 ± 0.0011	3204 ± 15<br>1581 ± 5.4<br>1484 ± 0.43<br>1388 ± 2.1<br>1233 ± 0.90<br>1097 ± 0.85<br>1005 ± 0.37<br>979 ± 2.5<br>838 ± 0.67<br>813 ± 1.4<br>738 ± 2.7
Pyrogallol (n = 4)	0.31 ± 0.027	1.4 ± 0.093	0.0130 ± 0.0012	3199 ± 29<br>1574 ± 5.5

				1375 ± 0.88<br>771 ± 1.0
Norepinephrine (n = 2)	0.47 ± 0.17	1.6 ± 0.15	0.0111 ± 0.011	3202 ± 10<br>1574 ± 8.6<br>1375 ± 0.81<br>781 ± 3.6
Epinephrine (n = 2)	0.022 ± 0.012	1.5 ± 0.053	0.0135 ± 0.0015	3220 ± 13<br>1597 ± 2.1<br>1521 ± 0.71<br>1361 ± 0.71<br>1297 ± 0.71<br>1241 ± 0.01<br>1155 ± 0.71<br>1097 ± 0.01<br>960 ± 0.01<br>941 ± 0.01<br>885 ± 0.01<br>768 ± 0.01
Caffeic acid (n = 3)	0.21 ± 0.077	1.5 ± 0.034	0.0152 ± 0.0031	3220 ± 7.2<br>1552 ± 1.0<br>1507 ± 0.54<br>1382 ± 0.27<br>1262 ± 0.61<br>1120 ± 0.52<br>975 ± 1.15<br>799 ± 2.0<br>710 ± 0.57
Dihydrocaffeic acid (n = 3)	0.077 ± 0.018	0.78 ± 0.088	0.0123 ± 0.00062	3337 ± 9.9<br>1552 ± 4.4<br>1394 ± 1.2<br>937 ± 1.4

				877 ± 1.4<br>799 ± 0.58
3,4-dihydroxy benzoic acid (n = 2)	0.11 ± 0.023	1.2 ± 0.24	0.0129 ± 0.00030	3226 ± 37<br>1559 ± 2.9<br>1372 ± 1.9<br>787 ± 0.13
Serotonin (n = 3)	0.48 ± 0.019	1.8 ± 0.17	0.0036 ± 0.00078	3207 ± 33<br>1580 ± 1.0<br>1351 ± 1.0<br>797 ± 1.0
DHI (n = 2)	2.55 ± 0.46	3.14 ± 0.67	0.0050 ± 0.000041	3218 ± 11<br>1568 ± 3.8<br>1353 ± 3.1<br>783 ± 1.9

**Supplementary Table S2 : pictures of melanin samples synthesized from pyrogallol, dopamine or DHI dispersed at varying concentrations** (Photographs by K.V.)					
Pyrogallol-based material					
	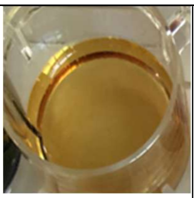 C = 472 μg/mL	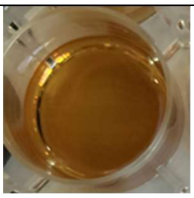 C = 810 μg/mL	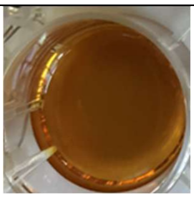 C = 1417 μg/mL	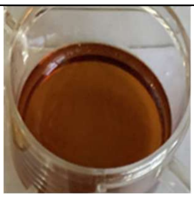 C = 2125 μg/mL	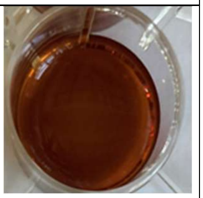 C = 2833 μg/mL
Dopamine-based material					
	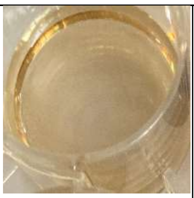 C = 132 μg/mL	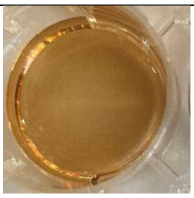 C = 362 μg/mL	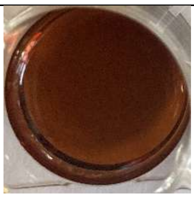 C = 925 μg/mL	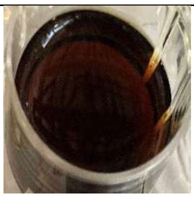 C = 1388 μg/mL	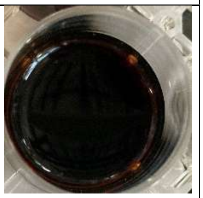 C = 2775 μg/mL
DHI-based material					
	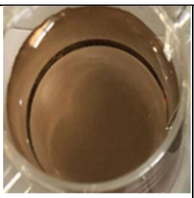 C = 46 μg/mL	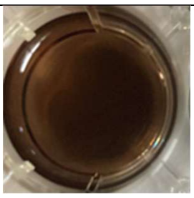 C = 90 μg/mL	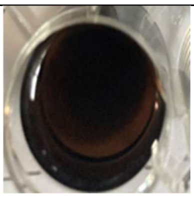 C = 175 μg/mL	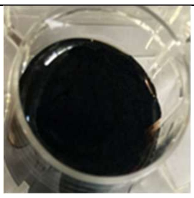 C = 560 μg/mL	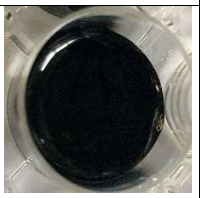 C = 2800 μg/mL

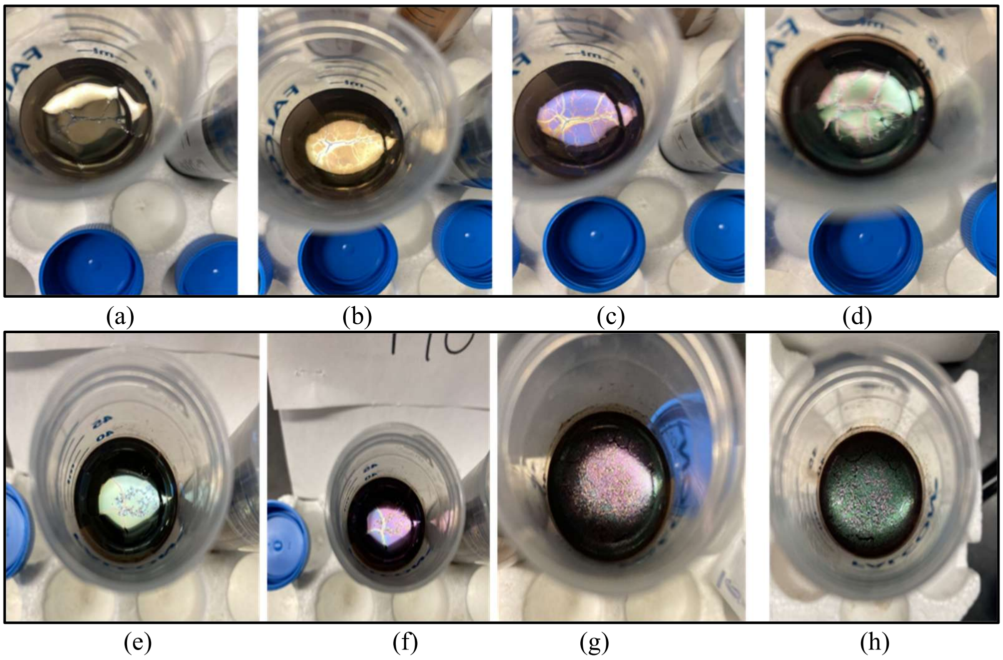

Supplementary Figure S1. Top row: photographs of the top view of a stagnant reaction mixture containing serotonin (see Section 2.1). (a) Perpendicular view after 24 hours of reaction, (b) angled view after 24 hours of reaction, (c) angled view after 26 hours of reaction and (d) angled view after 7 days of reaction. Bottom row: photographs of secondary film formation (see Section 2.1). (e) angled view after 2 days, (f) angled view after three days, (g) angled view after six days and (h) angled view after seven days of additional reaction.(Photographs by K.V.)

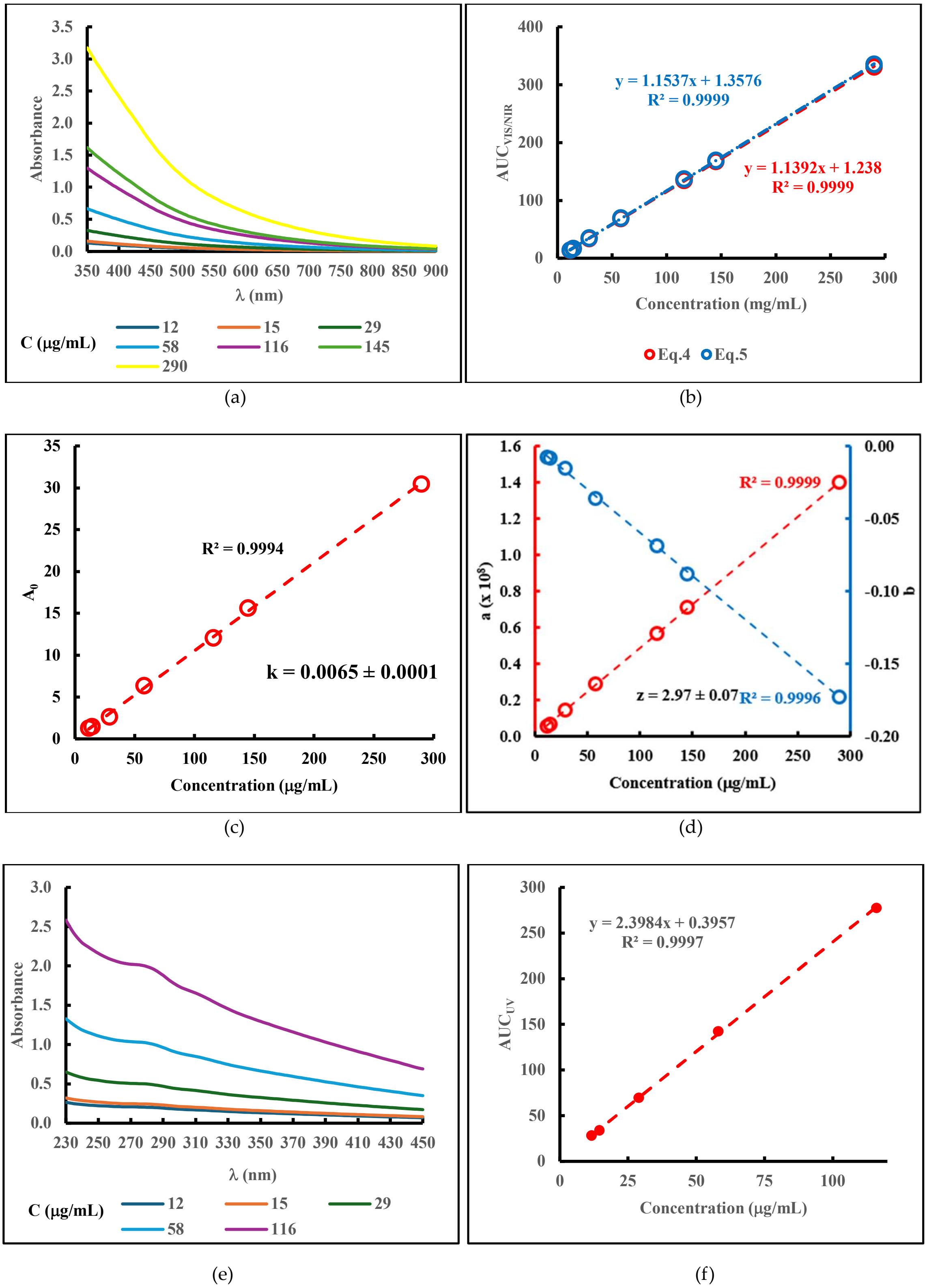

Supplementary Figure S2. (a) Absorbance profiles in vis/NIR region of L-DOPA based MN dispersed in water at varying concentrations. (b) Comparison of the values of $AUC_{vis/NIR}$ calculated using Equation (4) or Equation (5) as a

function of sample concentration. (c) Linear relationship between the value of A_0 from Equation (1) as a function of sample concentration and average value (± standard deviation; n = 7) of k from Equation (1). (d) Linear relationships between the values of a (red) or b (blue) from Equation (2) as a function of sample concentration and average value (± standard deviation; n = 7) of z from Equation (2). (e) UV absorbance profiles of L-DOPA based MN dispersed in water at varying concentrations. (f) Relationship between the values of AUC_{UV} calculated using Equation (6) and sample concentration.

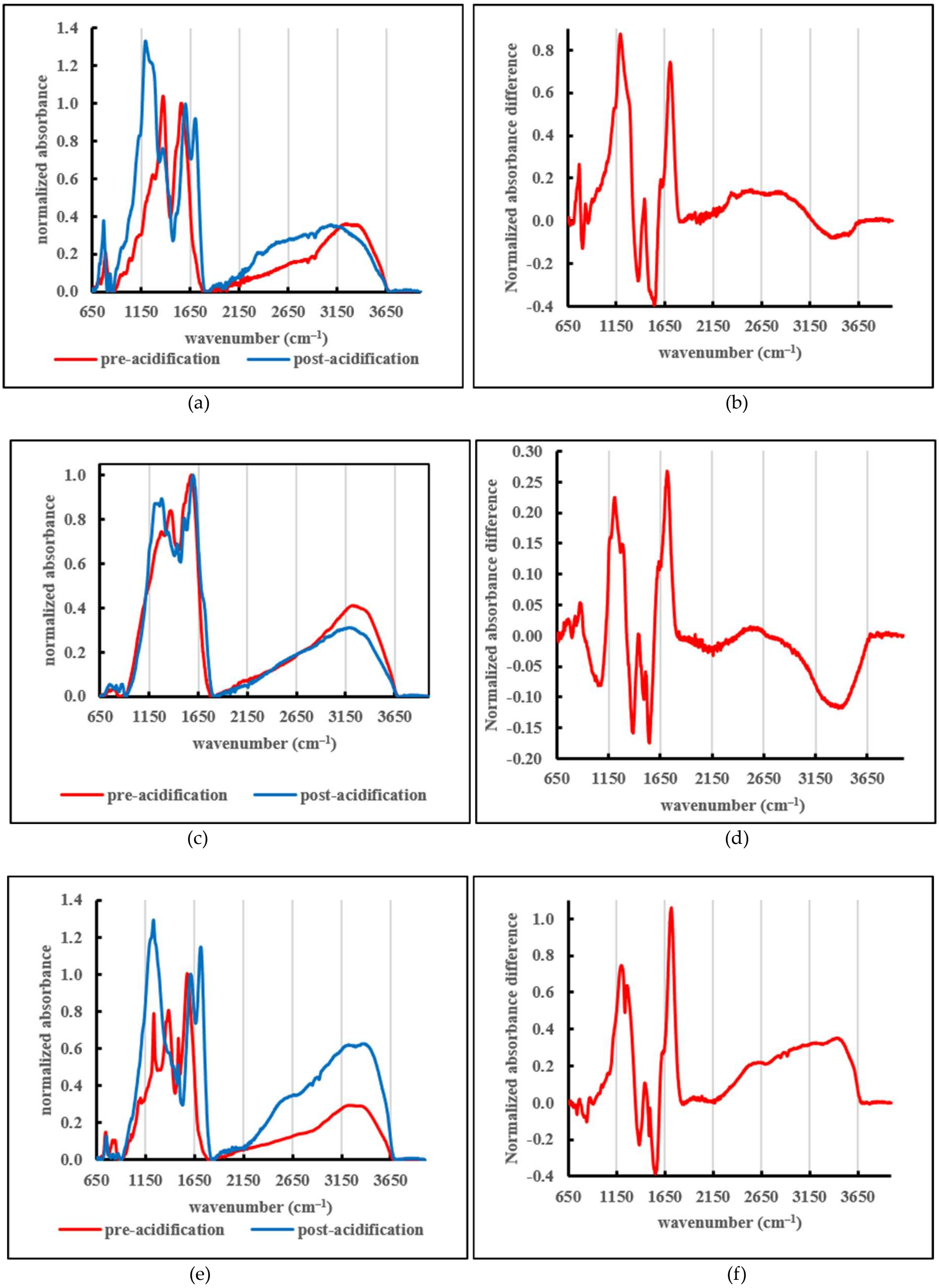

Supplementary Figure S3. (a) Normalized FT-IR spectra of 3,4-dihydroxybenzoic acid-based MN pre- and post-acidification. (b) Absorbance difference following subtraction of normalized FT-IR spectrum of 3,4-dihydroxybenzoic

acid-based MN pre-acidification, from the normalized FT-IR spectrum of the same material post-acidification. (c) Normalized FT-IR spectra of dopamine-based MN pre- and post-acidification. (d) Absorbance difference following subtraction of normalized FT-IR spectrum of dopamine-based MN pre-acidification, from the normalized FT-IR spectrum of the same material post-acidification. (e) Normalized FT-IR spectra of catechol-based MN pre- and post-acidification. (f) Absorbance difference following subtraction of normalized FT-IR spectrum of catechol-based MN pre-acidification, from the normalized FT-IR spectrum of the same material post-acidification.
